# Fungicide Sensitivity Profile of *Pyrenophora teres* f. *teres* in Field Population

**DOI:** 10.3390/jof10040260

**Published:** 2024-03-29

**Authors:** Regina Pütsepp, Andres Mäe, Lee Põllumaa, Liis Andresen, Riinu Kiiker

**Affiliations:** Centre of Estonian Rural Research and Knowledge, 48309 Jõgeva Alevik, Estonia; regina.pytsepp@metk.agri.ee (R.P.);

**Keywords:** net blotch, fungicide sensitivity, CYP51, *Cyp51A* promoter, SDH subunits, Cyt b, mating type

## Abstract

*Pyrenophora teres* f. *teres* (*Ptt*) is a severe pathogen to spring barley in Northern Europe. *Ptt* with relevant mutations in fungicide target proteins, sterol 14α-demethylase (CYP51A), cytochrome b (Cyt b), and succinate dehydrogenase (SDH) would put efficient disease control at risk. In the growing seasons of 2021 and 2022, 193 *Ptt* isolates from Estonia were analysed. In this study, mutation detection and in vitro fungicide sensitivity assays of single-spore isolates were carried out. Reduced sensitivity phenotype to mefentrifluconazole was evident in *Ptt* isolates with a F489L mutation in CYP51A or with 129 bp insert in the *Cyp51A* gene-promoter region. However, sensitivity to a prothioconazole-desthio remained high regardless of these molecular changes. The *Ptt* population was mostly sensitive to bixafen, fluxapyroxad, pyraclostrobin, and azoxystrobin. The sensitivity of fluxapyroxad and bixafen has been affected by two mutations, C-S135R and D-H134R, found in SDH subunits. The F129L mutation in Cyt b influenced azoxystrobin but not pyraclostrobin sensitivity. In total, 30 isolates from five fields had relevant mutations in three target protein genes simultaneously. Most of these isolates had a reduced sensitivity phenotype to mefentrifluconazole, fluxapyroxad, and azoxystrobin, while sensitivity to other tested fungicides remained high. Furthermore, possible sexual reproduction may enhance the pathogen’s fitness and help it adapt to fungicides.

## 1. Introduction

Barley is one of the oldest cultivated crops, which is resilient in different growing conditions and under stress factors. It is a valuable crop for multiple uses, e.g., alcohol production, feed for livestock, and human consumption [1]. Spring barley is the second most cultivated crop in Estonia because it fits well in the crop rotation and has satisfactory yields under regional climatic and agricultural conditions with low fertilizer and pesticide input [2].

Net blotch is a common barley disease caused by two forms of *Pyrenophora teres*: net form is caused by *P. teres* f. *teres* (*Ptt*) and spot form is caused by *P. teres* f. *maculata* (*Ptm*) [3]. The net form net blotch is the most prevalent foliar disease, causing substantial loss of spring barley in Estonia, as well as in other Northern European countries, where the climatic conditions are favourable for the development of the disease and pathogen spread, susceptible hosts are grown, and primary inoculum is not fully diminished [4,5,6,7,8].

The life cycle of *Ptt* involves both asexual and sexual stages. The asexual lifecycle includes rapid reproduction and conidial spread by wind and rain to neighbouring plants and fields, as well as overwintering of the pathogen as mycelium in seed or in crop debris. For sexual reproduction, the opposite mating types (MAT-1 and MAT-2) of the pathogen need to meet on the host [9]. The sexual fruiting bodies, pseudothecia, are formed on the barley straw after harvest in autumn, enabling pathogen survival through winter [10]. Sexual recombination increases genetic diversity and virulence variation of the pathogen, probably improving the adaptation and fitness of the pathogen [11]. Pathogen populations with mixed reproduction system are expected to quickly adapt to fungicides with a single mode of action [11]. It is probable that sexual reproduction occurs at a high frequency within the worldwide population of *P. teres*, but the dynamics are influenced by local agronomic and environmental conditions [12].

The primary inoculum sources early in the season are the mature ascospores discharged from the asci in pseudothecia, as well as seed-borne mycelium and conidia released from the barley straws dispersed by wind to nearby fields [10,12,13]. Sowing healthy seeds and practicing longer crop rotation and soil tillage are the main modes of destruction of primary inoculum [13]. Spring barley cultivars bred in Europe are variable in net blotch resistance, but resistance breeding is more relevant than ever [14]. However, reduced and no-tillage cultivation systems are becoming more popular among farmers around the world, making disease management more demanding [15]. Optimizing fertilizer and fungicide applications would increase the crop quality and yield of barley. In Estonia, seed treatment and two foliar applications of fungicide per growing season tend to give the best outcome [7,8]. Optimal disease control with fungicides can give a substantial barley yield increase under high disease pressure [5,6]. However, under continuous selection pressure with fungicide application, a fungal population can evolve toward reduced sensitivity, and the proportion of insensitive phenotypes may reach a level where satisfactory disease control is no longer achieved [16]. It is also evident that selection for fungicide insensitivity in the pathogen population is increased with higher fungicide dosage due to survival and reproduction of the fittest genotypes [16].

Quinone outside inhibitor (QoI), succinate dehydrogenase inhibitor (SDHI), and demethylation inhibitor (DMI) fungicides are widely used in cereal plant protection. The selection of fungicides allowed in Estonia is limited, and the four most popular fungicides (DMIs prothioconazole and tebuconazole, amine fungicide spiroxamine, and QoI pyraclostrobin) constitute over 55% of the total amount of fungicides applied in agriculture [17]. Due to selection pressure, the loss of QoI, SDHI, and DMI sensitivity has spread in *Ptt* populations in several European countries, Canada, and Australia [18,19,20,21,22,23].

Three amino acid substitutions (e.g., G143A, G137R, F129L) have been detected in the cytochrome b (Cyt b) in plant pathogens with insensitivity to QoI-s. Mutations F129L and G137R have a low to moderate effect on QoI insensitivity in the pathogen´s population, and the field performance of QoI fungicides still remains good in Europe [24]. However, mutation G143A in Cyt b of *Pyrenophora tritici-repentis*, *Blumeria graminis* f. sp. *tritici*, and *Zymoseptoria tritici* has a strong effect on sensitivity reduction [25,26,27]. Until recently, only the F129L mutation in Cyt b had been reported for *P. teres* [20,22,23]. In several phytopathogens (e.g., *Ptt*, *Alternaria* spp., *Puccinia* spp.), a nucleotide mutation at a position near the exon/intron junction, such as that which leads to the G143A substitution, would affect the splicing process, and non-functional Cyt b proteins would be produced. It would reduce the fitness and survival of the pathogen markedly [28,29]. There was a rare finding of a *Ptt* field isolate named ISO-2210 from Denmark in 2019, which was highly insensitive to QoI fungicides due to G143A mutation in Cyt b, which was assumed to be a chimer of *P. teres* and *P. tritici-repentis* [27].

Reduced sensitivity to SDHI fungicides in *Ptt* populations is related to target-site mutations in succinate dehydrogenase (SDH) subunits, the most significant being C-G79R, C-H134R, C-S135R, D-D124E, and D-H134R [21]. The first findings of SDHI insensitive *Ptt* isolates, which had B-H277Y substitution, were identified in Germany in 2012, and several mutations in SDH subunits have occurred since then [21]. In Eastern Europe, *Ptt* populations have still high sensitivity to SDHIs, while since 2017, the sensitivity has been reduced in Western Europe [30].

Three primary mechanisms of DMI insensitivity are (i) mutations in the target-encoding sterol 14α-demethylase (*Cyp51*) gene, (ii) over-expression of the target *Cyp51* gene, and (iii) increased efflux caused by the over-expression of genes encoding membrane transporters. Insensitivity levels are often determined by combinations of these mechanisms [31]. The ascomycete species genome has undergone several *Cyp51* gene duplication and divergence events; for example, *Pyrenophora* spp. have two paralogs (*Cyp51A* and *Cyp51B*) and *Aspergillus* spp. and *Fusarium* spp. have three paralogs (*Cyp51A*, *Cyp51B*, and *Cyp51C*) [19,32]. Species with multiple copies of the *Cyp51* gene are intrinsically less sensitive to some DMI fungicides, and mutations conferring acquired insensitivity to effective azoles are usually restricted to one paralogue, most often *Cyp51A* [33]. Mair et al. [19] showed that *Ptt* DMI insensitivity was correlated with two genetic modifications, an amino acid change F489L in the CYP51A protein, and overexpression of *Cyp51A* and *Cyp51B* genes. *Ptm* isolates carrying both a 134 bp insertion element in the *Cyp51* promoter and an F489L mutation in CYP51A displayed the highly DMI-insensitive phenotype [34].

In Estonia, fungicide sensitivity in the *Ptt* population is unknown, and testing for fungicide efficacy in field trials is scarce. Being aware of the pathogen sensitivity to fungicides is essential to execute effective and knowledge-based net blotch management strategies and to prolong the effective lifetime of fungicides. Hopefully, it also increases crop yields, additionally taking into account cultivar selection, tillage, optimal fertilization, water supply, climatic conditions, etc.

In this first comprehensive study of the Estonian *Ptt* population, we focused on the current status of fungicide sensitivity and its association with relevant SNPs in target protein-coding gene sequences linked to fungicide insensitivity [19,21,34,35,36]. Therefore, the objectives of this research were to assess the Estonian *Ptt* population for (i) fungicide sensitivity level in vitro microtiter plate assays and subsequently associate insensitivity with (ii) Cyt b mutations, (iii) SDH subunit mutations, (iv) molecular changes in the *Cyp51A* gene and promoter region; and (v) potential sexual recombination by the analysing mating type prevalence rate.

## 2. Materials and Methods

### 2.1. Field Sampling and Fungal Isolates

Leaves with net form net blotch symptoms were collected from commercial spring barley fields in the years 2021 and 2022 across Estonia. The crop had been treated once or twice with foliar fungicides (DMIs, DMI, and SDHI mixture or DMI and QoI mixture). The leaves were dried at room temperature (21 °C) and were then surface sterilized with a 70% ethanol solution for 2 min and rinsed with distilled water twice for 2 min. Leaf sections of 10 cm were then placed on 1% water agar Petri dishes (diam. 14 cm) and incubated for 3–5 days (at room temperature in dark). When conidiophores were visible, single conidia were picked with a sterile needle and placed on potato dextrose agar (PDA; VWR International, Leuven, Belgium) plates amended with 0.1 mg/mL streptomycin (AppliChem GmbH, Darmstadt, Germany). The pure culture isolates were incubated at 22 °C in a 12 h white (TL5 54W/840 HO light tubes by Philips, Amsterdam, the Netherlands)/17 °C 12 h dark photoperiod for 1–2 weeks depending on the growth speed of the isolates. A total of 60 and 210 *Ptt* isolates were acquired in years 2021 and 2022, respectively.

### 2.2. DNA Extraction and Species Determination

For DNA extraction, the mycelia of each isolate were collected with the sterile inoculation loop from the pure culture isolate plates, lyophilized with a FreeZone 2.5 Liter-50C Benchtop Freeze Dryer (Labconco, Kansas City, MO, USA), and homogenized with a TissueLyzer (Qiagen, Düsseldorf, Germany) in 2 mL tubes with Retsch balls. For further isolation, a QIAcube^®^ HT DNA extractor (Qiagen, Düsseldorf, Germany) and a DNeasy mericon 96 QIAcube HT Kit were used according to the manufacturer’s instructions [37]. After DNA isolation, the *Ptt* isolates were confirmed with PCR and Sanger sequencing using ITS1 and ITS4 primers according to White et al. [38] and a *Ptt* form-specific assay with primers according to Knight et al. [35] (Table 1). PCR amplification was performed with a total volume of 20 µL mix consisting of 2 µL of 10× DreamTaq Green PCR buffer (Thermo Fisher Scientific, Waltham, MA, USA), 2 µL 10× GC-rich Enhancer (Solis BioDyne, Tartu, Estonia), 100 µM of each dNTP, 0.4 µM of specific forward and reverse primers (Table 1), 1 unit DreamTaq Hot Start DNA Polymerase (Thermo Fisher Scientific, Waltham, MA, United States), 10 ng of genomic DNA, and the remaining amount of MilliQ water (Merck KGaA, Darmstadt, Germany). The amplification parameters were as follows: an initial denaturation for 3 min at 95 °C, followed by 35 cycles of denaturation at 95 °C for 30 s, annealing at 55 °C (ITS) or 61 °C (*Ptt* form-specific) for 30 s, and extension at 72 °C for 30 s, with final extension at 72 °C for 5 min. All the amplifications were performed in a Mastercycler nexus (Eppendorf, Hamburg, Germany). The PCR products of the ITS region were sequenced using an Applied Biosystems 3730 DNA Analyzer (Thermo Fisher Scientific, Waltham, MA, USA) at the Institute of Genomics Core Facility, University of Tartu (Estonia). The sequences obtained were analysed using blastn search tools (https://blast.ncbi.nlm.nih.gov/Blast.cgi (accessed 21 December 2023)) available at NCBI. The amplicons (213 bp) for the *Ptt* form-specific control were separated by 1.5% agarose gel electrophoresis in 1xTAE (Tris-acetate-EDTA) buffer (pH 8.0).

### 2.3. In Vitro Fungicide Sensitivity Assay

**Figure 1:**
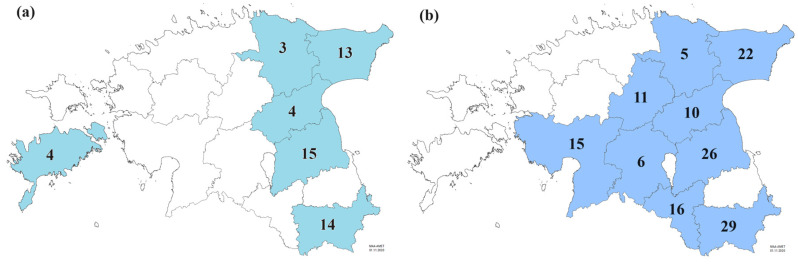
Number of *Pyrenophora teres* f. *teres* isolates analysed in 2021 (**a**) and 2022 (**b**) collected from different Estonian counties (modified maps of administrative units from Estonian Land Board [40]).

A total of 53 *Ptt* isolates from 2021 and 140 isolates from 2022 were selected randomly to cover a wide selection of commercial fields (12 fields in 2021, 23 fields in 2022) to perform fungicide sensitivity assays and further molecular analysis (Figure 1). The selected isolates were grown on POA medium (20 g of grinded green peanut leaves, 6 g whole-grain oat flour, 6 g agar, filled up to 400 mL of water) amended with 0.1 mg/mL streptomycin on Petri plates (diam. 4.5 cm). The isolates were exposed to periods of white light (12 h at 22 °C) and darkness (12 h at 17 °C) for two weeks. For further preservation, the hyphae and conidia were suspended in 20% sterile glycerol and stored at ultra-low temperature—82 °C. For the sensitivity assay, the fungal mycelium was dislodged from the Petri dishes with a sterile inoculation loop and suspended in 5 mL of 2xYBA medium (8 g Bacto Yeast Extract, 8 g Bacto Tryptone, 16 g anhydrous sodium acetate, 400 mL of distilled water). The Petri dishes were washed with about 2–3 mL of 2xYBA medium and pipetted into the same tube as the mycelium. The tubes were vortexed on a Multi Reax (Heidolph Instruments, Schwabach, Germany) at 2000 rpm for 15 min. The vortexed samples were filtered through pluriStrainer 200 µm mesh (pluriSelect Life Science, Leipzig, Germany) into a sterile 15 mL Falcon tube. Because the spores did not emerge sufficiently, pieces of hyphae were then counted and adjusted to a density of 4 × 10^3^ pcs/mL. Each fungicide technical active ingredient (Dr. Ehrenstorfer GmbH, Augsburg, Germany) was dissolved in 80% ethanol, and the desired dilutions were prepared in sterile deionised water. Approximately 50 µL of fungicide solution and 50 µL of hyphae suspension were mixed in 96-well microtiter plates. The final concentrations of mefentrifluconazole and bixafen were 0, 0.01, 0.04, 0.12, 0.37, 1.11, 3.33, and 10 mg/L; of azoxystrobin, pyraclostrobin, and prothioconazole-desthio were 0, 0.008, 0.025, 0.07, 0.22, 0.67, 2, and 6 mg/L; and of fluxapyroxad were 0, 0.003, 0.01, 0.037, 0.11, 0.33, 1, and 3 mg/L on the microtiter plates. For each isolate, three replicates were used. A negative control with each fungicide solution and 2xYBA medium without hyphae suspension was added to the assay. The microtiter plates were wrapped in aluminium foil and incubated at 20 °C in darkness. After 5 days, the optical density (OD_405_) was measured with a Tecan Sunrise™ absorbance reader (Tecan, Männedorf, Switzerland). The values were corrected by comparison with the negative controls, and the EC_50_ was determined by non-linear regression (curve-fit) with a variable slope using GraphPad Prism 9.4.1 (GraphPad Software, San Diego, CA, United States). The *Ptt* isolates were grouped according to relevant amino acid substitutions in fungicide target molecules and in vitro fungicide sensitivity. According to mefentrifluconazole and prothioconazole-desthio sensitivity results, the *Ptt* isolates were grouped as follows: EC_50_ < 1 mg/L as sensitive, EC_50_ = 1–5 mg/L as reduced sensitivity, EC_50_ > 5 mg/L as insensitive. The *Ptt* isolates were grouped based on fluxapyroxad, and the bixafen sensitivity results were as follows: EC_50_ < 0.1 mg/L as sensitive, EC_50_ = 0.1–1 mg/L as reduced sensitivity, EC_50_ > 1 mg/L as insensitive. The *Ptt* isolates were grouped based on pyraclostrobin, and the azoxystrobin sensitivity results were as follows: EC_50_ < 0.01 mg/L as sensitive, EC_50_ = 0.01–1 mg/L as reduced sensitivity, EC_50_ > 1 mg/L as insensitive.

### 2.4. Molecular Analysis of Fungicide Target Site Protein Genes, Cyp51A Promoter Region, and Mating Type

To assess different target site mutations and insertions in the *Cyp51A* promoter region, PCR amplifications were performed for the *Cyp51A*, *sdhB*, *sdhC*, *sdhD*, and *cyt b* gene sequences and the *Cyp51A* promoter with the selected primers indicated in Table 1. Based on the genome assembly of the *Ptt* isolate W1-1 (GenBank accession GCA_900232045.3), new primers were designed for detecting G137R mutation in Cyt b using Primer3 software v. 0.4.0 [41,42]. The sequence similarity and specificity were verified using the Primer-BLAST search tool (https://www.ncbi.nlm.nih.gov/tools/primer-blast/index.cgi?LINK_LOC=BlastHome (accessed 1 June 2023)) of the NCBI GenBank Standard databases. PCR optimisation and specificity assessment was then performed using target (*Ptt*) and non-target (*Z. tritici, Ramularia collo-cygni*) isolate DNA. PCR amplification was performed with a total volume of 20 µL mix consisting of 2 µL of 10× DreamTaq Green PCR buffer (Thermo Fisher Scientific, Waltham, MA, USA), 2 µL 10× GC-rich Enhancer (Solis BioDyne, Tartu, Estonia), 100 µM of each dNTP, 0.4 µM of specific forward and reverse primers (Table 1), 1 unit DreamTaq Hot Start DNA Polymerase (Thermo Fisher Scientific, Waltham, MA, United States), 10 ng of genomic DNA, and the remaining amount of MilliQ water. The amplification parameters for the *Cyp51A* gene and *Cyp51A* promoter region were as follows: an initial denaturation for 3 min at 95 °C, followed by 35 cycles of denaturation at 95 °C for 30 s, annealing at 55 °C (*Cyp51A* upstream region), 58 °C (*Cyp51A* promoter), or 62 °C (*Cyp51A*) for 30 s, and extension at 72 °C for 2 min, with final extension at 72 °C for 5 min. The amplification parameters for *sdhB*, *sdhC*, and *sdhD* were as follows: an initial denaturation for 3 min at 95 °C, followed by 35 cycles of denaturation at 95 °C for 30 s, annealing at 60 °C (*sdhC* and *sdhD*) or 63 °C (*sdhB*) for 30 s, and extension at 72 °C for 1 min, with final extension at 72 °C for 5 min. The *Cyt b* amplification parameters were as follows: an initial denaturation for 3 min at 95 °C, followed by 35 cycles of denaturation at 95 °C for 20 s, annealing at 58 °C for 25 s, and extension at 72 °C for 30 s, with final extension at 72 °C for 5 min. All the amplifications were performed in a Mastercycler nexus (Eppendorf, Hamburg, Germany). The PCR products were sequenced using an Applied Biosystems 3730 DNA Analyzer (Thermo Fisher Scientific, Waltham, MA, USA) at the Institute of Genomics Core Facility, University of Tartu (Estonia). The sequences obtained were analysed, and the target-site mutations were identified using blastn and blastx search tools (https://blast.ncbi.nlm.nih.gov/Blast.cgi (accessed 21 December 2023)) available at NCBI. We focused on previously published relevant SNPs in the target protein-coding gene sequences associated with fungicide insensitivity [19,21,34,35,36]. The representative sequences of the fungicide target protein genes of the studied *Ptt* isolates are deposited in NCBI GenBank with accession numbers OR530176, OR761967 to OR761973, and OR777246 to OR777248 (Table S1).

To determine the mating type of the isolates, PCR amplification was performed in a multiplex of MAT-1 and MAT-2 primers [39] (Table 1) with the following parameters: an initial denaturation for 3 min at 95 °C, followed by 34 cycles of denaturation at 95 °C for 50 s, annealing at 60 °C for 30 s, and extension at 72 °C for 1 min, with final extension at 72 °C for 7 min. The amplicons, 1143 bp for MAT-1 and 1421 bp for MAT-2, were separated by 1% agarose gel electrophoresis in 1xTAE (Tris-acetate-EDTA) buffer (pH 8.0).

### 2.5. Statistical Analysis

The statistical analysis was performed in GraphPad Prism 9.4.1 (GraphPad Software, San Diego, CA, USA). The EC_50_ values were log_10_-transformed prior to the statistical analysis. An unpaired *t*-test with Welch’s correction was applied to compare the *Ptt* fungicide sensitivity between the years and between the mutated and wild-type isolates (α = 0.05). A Kruskal–Wallis test with Dunn’s multiple comparison test was performed to compare the three groups (α = 0.05). The mating type distribution within the counties and years was analysed with a binomial test to find if discrepancy is significant (*p* < 0.05) from the MAT-1 and MAT-2 1:1 distribution. The figures were visualized using Igor Pro 6.36 (WaveMetrics, Portland, OR, USA). The *Cyp51A* gene-promoter sequence alignment was visualized using Jalview 2.11.3.0 [43].

## 3. Results

### 3.1. Pyrenophora teres *f.* teres Sensitivity to DMI Fungicides

**Figure 2:**
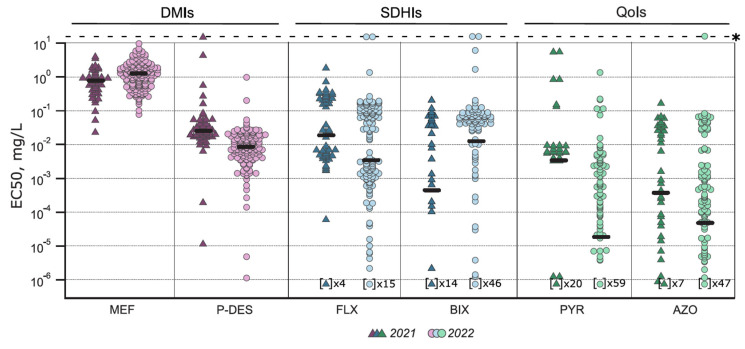
Fungicide sensitivity of *Pyrenophora teres* f. *teres* population in 2021 and 2022. *Ptt* isolates were tested in microtiter plate sensitivity assay against six fungicides: demethylation inhibitors (DMIs) mefentrifluconazole (MEF) and prothioconazole-desthio (P-DES), succinate dehydrogenase inhibitors (SDHIs) fluxapyroxad (FLX) and bixafen (BIX), quinone outside inhibitors (QoIs) pyraclostrobin (PYR) and azoxystrobin (AZO). Median EC_50_ values are marked with thick horizontal line and insensitive isolates with asterisk. Note that the EC_50_ value in the case of number of isolates indicated in brackets remained within the limit of quantification (10^−6^ mg/L) in the experiments.

**Figure 3:**
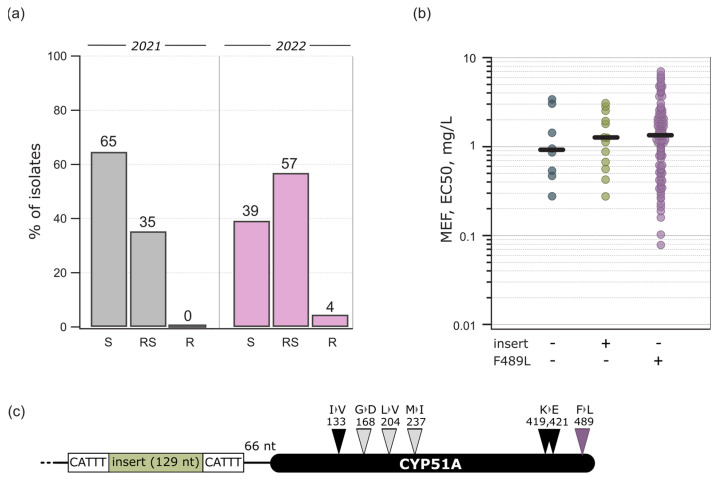
Mefentrifluconazole sensitivity and variations in its target CYP51A. (**a**) Percentage of sensitive (S, EC_50_ < 1 mg/L), reduced sensitivity (RS, EC_50_ = 1–5 mg/L), and insensitive isolates (R, EC_50_ > 5 mg/L) in 2021 and 2022; (**b**) distribution of *Ptt* isolates according to F489L mutation in CYP51A and 129 bp insert in *Cyp51A* promoter in 2022; (**c**) insert position in *Cyp51A* promoter and CYP51A with the frequent (dark) and rare (light) mutations.

The *Ptt* population sensitivity to a DMI fungicide mefentrifluconazole was low. Sensitivity to mefentrifluconazole was significantly different between the isolates collected in 2021 and 2022 (*p* < 0.001, t = 4.135, df = 128.9): the median EC_50_ were 0.812 mg/L and 1.329 mg/L, respectively (Figure 2). In 2021, 65% of the isolates were sensitive to mefentrifluconazole (EC_50_ < 1 mg/L) and 35% had a reduced sensitivity phenotype (Figure 3a). Mefentrifluconazole sensitivity reduction was more prominent in 2022, where 4% of the isolates had an insensitive phenotype, 57% had a reduced sensitivity phenotype, and 39% were sensitive (Figure 3a). On the contrary to mefentrifluconazole, the *Ptt* population was sensitive to another tested DMI prothioconazole derivate, prothioconazole-desthio: the median EC_50_ was 0.026 mg/L in 2021 and 0.009 mg/L in 2022 (Figure 2). In addition, two exclusive isolates with reduced sensitivity to both DMIs were found.

The most frequent mutations in CYP51A were I133V, K419E, K421E, and F489L (Table 2, Figure 3). Two different codons gave rise to the phenylalanine to leucine amino acid change in amino acid position 489 from the wild-type TTC to either CTC or TTA (Table S2). All the *Ptt* isolates from 2021 (haplotypes PttEE-A3 and 21PttEE-15) and 79% from 2022 had these four mutations in CYP51A (haplotypes PttEE-A3 and PttEE-A4; Table 2). Only a few isolates had other rare mutations, for instance, G168D (haplotypes PttEE-4507 and PttEE-4506), L204V (haplotype 21PttEE-15), and M237I (haplotype PttEE-14).

The *Cyp51A* gene-promoter region was mostly wild-type. However, 16 isolates in 2022 had an identical 129 bp insert sequence with 5 bp distinct repeats on both sides of the promoter region (Figure 3c and Figure S1; GenBank accession no. OR530176). None of the isolates had F489L mutation in CYP51A together with an insert in the gene-promoter region. Among the isolates collected in 2022, the mefentrifluconazole sensitivity was highest in the isolates with phenylalanine in position 489 in CYP51A and a wild-type promoter (median EC_50_ = 0.927 mg/L), followed by isolates with promoter insert (median EC_50_ = 1.15 mg/L) and F489L-mutated isolates (median EC_50_ = 1.386 mg/L) with reduced sensitivity (Figure 3b). The difference between these groups of isolates was not significant (*p* = 0.66). However, the outcome of the prothioconazole-desthio sensitivity analysis was opposite to the mefentrifluconazole analysis. The EC_50_ of prothioconazole-desthio differentiated significantly between these groups of isolates (*p* < 0.001, H = 47.3, df = 2), with the sensitivity being the highest in the F489L-mutated isolates (median EC_50_ = 0.007 mg/L) and comparable between isolates with an unmutated 489 position in CYP51A together with a wild-type promoter and inserted promoter region isolates. The median EC_50_ values were 0.024 mg/L and 0.025 mg/L, respectively. However, all the isolates except for two were completely sensitive to prothioconazole-desthio, and these molecular changes did not reduce the sensitivity.

### 3.2. Pyrenophora teres *f.* teres Sensitivity to SDHI Fungicides

Fluxapyroxad and bixafen sensitivity was analysed in the *Ptt* population. In 2021, the *Ptt* population was more sensitive to bixafen than fluxapyroxad and vice versa in 2022 (Figure 2). The fluxapyroxad sensitivity was significantly (*p* = 0.03) lower in 2021 compared to 2022 (t = 2.105, df = 60.63), and the median EC_50_ values were 0.02 mg/L and 0.004 mg/L, respectively (Figure 2). In total, nineteen isolates in 2021 (36%) and thirty-one in 2022 (22%) had a reduced sensitivity phenotype (EC_50_ = 0.1–1 mg/L) to fluxapyroxad. Only 2% of the isolates in both years were insensitive (EC_50_ > 1 mg/L) (Table 3). The shift in bixafen sensitivity between the years was not significant, and median EC_50_ values were 4.67 × 10^−4^ mg/L and 0.011 mg/L in 2021 and 2022, respectively (Figure 2). The frequency of isolates with reduced sensitivity phenotype to bixafen was comparable in 2021 and 2022 (Table 3). Insensitive phenotypes to bixafen occurred only in 2022 in four isolates (3%).

SDH subunits B, C, and D were sequenced for identifying mutations involved in SDHI sensitivity change. B-H277Y was missing in the Estonian *Ptt* population (Table 3). C-S135R was present in 16 isolates from 2021 (30%) and 19 isolates from 2022 (14%) (Table 3). It was a surprise to detect 43 isolates with amino acid change D-H134R in 2022 (31%), as no mutations in SDH-D were found in 2021 (Table 3).

**Figure 4:**
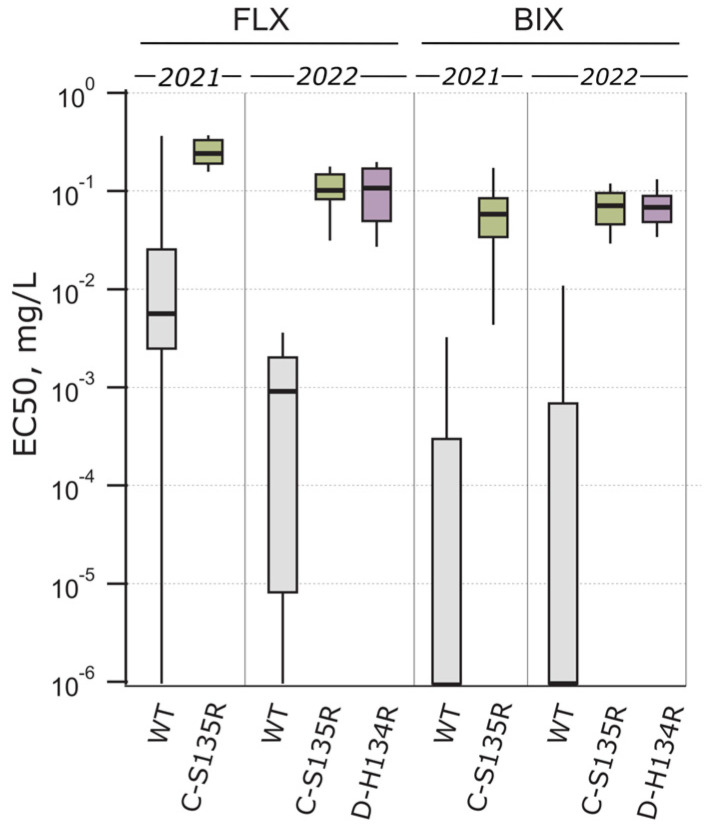
Fluxapyroxad (FLX) and bixafen (BIX) sensitivity and their dependence on SDH subunit mutations C-S135R and D-H134R. Box plot boundaries were set to 25th percentile and 75th percentile for the box, while lower and upper whiskers extend to 10th and 90th percentile, respectively.

The C-S135R and D-H134R mutations in the SDH subunits were mainly responsible for the sensitivity decline of fluxapyroxad and bixafen in the *Ptt* population. The wild-type and C-S135R-mutated isolates differentiated significantly in fluxapyroxad (*p* = 0.012, t = 2.615, df = 43.13) and bixafen sensitivity (*p* < 0.001, t = 4.447, df = 13.04) in 2021 (Figure 4). Additionally, in 2022, the fluxapyroxad (*p* < 0.001, H = 96.97, df = 2) and bixafen sensitivity (*p* < 0.001, H = 87.38, df = 2) differentiated significantly between the wild-type and C-S135R/D-H134R-mutated isolates (Figure 4). The fluxapyroxad sensitivity was significantly lower in 2021 among the C-S135R-mutated isolates compared to 2022 (*p* < 0.001, t = 6.909, df = 23.85), with the EC_50_ ranging from 0.137 to 0.365 mg/L (median EC_50_ = 0.238 mg/L) in 2021 and from 0.026 to 0.174 mg/L (median EC_50_ = 0.098 mg/L) in 2022. However, the fluxapyroxad sensitivity of the D-H134R-mutated isolates was comparable with the C-S135R-mutated isolates in 2022, with the EC_50_ ranging from 0.016 to 0.28 mg/L (median EC_50_ = 0.08 mg/L). In general, the bixafen sensitivity was comparable in the SDH-mutated isolates in the two study years, with the EC_50_ ranging from 0.001 to 0.216 mg/L (median EC_50_ = 0.058 mg/L) in 2021 and 0.015 to 0.118 mg/L (median EC_50_ = 0.07 mg/L) in 2022 for the C-S135R-mutated isolates and 0.027 to 0.212 mg/L (median EC_50_ = 0.067 mg/L) for the D-H134R-mutated isolates in 2022.

### 3.3. Pyrenophora teres *f.* teres Sensitivity to QoI Fungicides

The *Ptt* population was mostly sensitive to both tested QoI active ingredients, azoxystrobin and pyraclostrobin (Figure 2). The median EC_50_ for pyraclostrobin were 0.004 mg/L and 1.94 × 10^−5^ mg/L in 2021 and 2022, respectively. However, there were eight (18%) and nine (7%) reduced sensitivity phenotype isolates (EC_50_ = 0.01–1 mg/L) in 2021 and 2022, respectively (Table 3). Only two isolates (4%) in 2021 and one isolate (1%) in 2022 were insensitive to pyraclostrobin (EC_50_ > 1 mg/L) (Table 3). Sensitivity to azoxystrobin was also high, and the median EC_50_ was less than 0.001 mg/L in both years. However, fifteen isolates (37%) in 2021 and nineteen isolates (14%) in 2022 had a reduced sensitivity phenotype (EC_50_ = 0.01–1 mg/L), and only one isolate had an insensitive phenotype to azoxystrobin (EC_50_ > 1 mg/L) in 2022 (Table 3). All these isolates with an insensitive phenotype to pyraclostrobin or azoxystrobin had wild-type target protein Cyt b, so in this case, other mechanisms should be involved.

**Figure 5:**
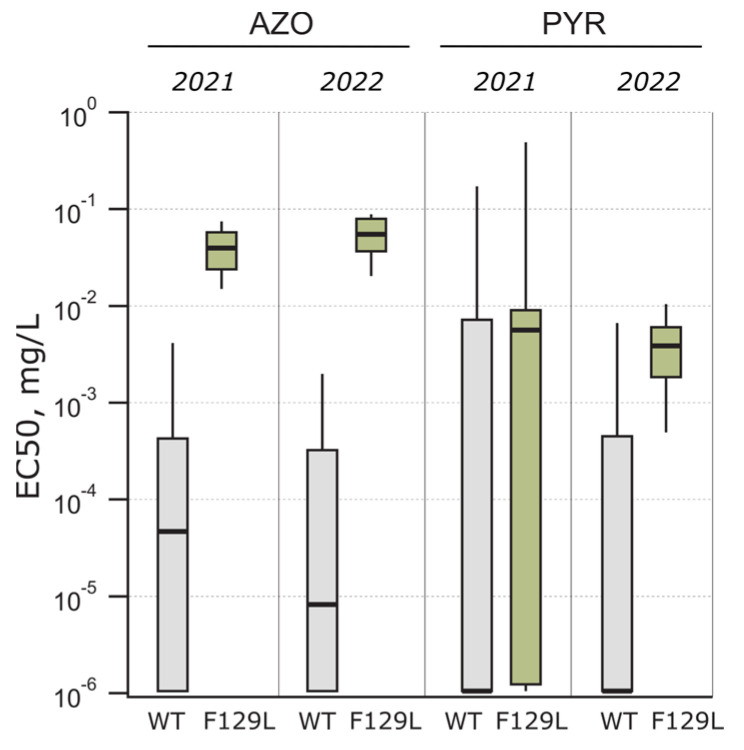
Azoxystrobin (AZO) and pyraclostrobin (PYR) sensitivity and their dependence on mutation F129L in Cyt b. Box plot boundaries were set to 25th percentile and 75th percentile for the box, while lower and upper whiskers extend to 10th and 90th percentile, respectively.

The *Cyt b* gene was amplified with two primer pairs to detect F129L and G137R mutations associated with QoI sensitivity in the *Ptt* isolates. In total, 16 and 17 *Ptt* isolates had an F129L mutation in Cyt b in 2021 and 2022, respectively (Table 3). At the same time, the G137R mutation was absent in Cyt b. An analysis revealed that all the isolates with the F129L mutation in Cyt b had azoxystrobin EC_50_ values higher than 0.01 mg/L. There was a significant difference (*p* < 0.001) between the wild-type and F129L-mutated isolates in azoxystrobin sensitivity in both years (2021: t = 3.646, df = 37.92; 2022: t = 11.49, df = 14.52) (Figure 5). The azoxystrobin sensitivity was significantly higher in 2021 among the F129L-mutated isolates compared to 2022 (*p* = 0.009, t = 2.802, df = 26.78), with the EC_50_ ranging from 0.012 to 0.071 mg/L (median EC_50_ = 0.036 mg/L) in 2021 and 0.029 to 0.088 mg/L (median EC_50_ = 0.05 mg/L) in 2022. However, there was no difference (*p* = 0.2) between the Cyt b-mutated and wild-type isolates in pyraclostrobin sensitivity in either year, and the pyraclostrobin sensitivity was highly variable (Figure 5).

### 3.4. Isolates with Relevant Mutations in All Three Target Site Proteins

Furthermore, 16 isolates in 2021 from two fields and 14 isolates in 2022 from three fields had a PttEE-A3 or PttEE-A4 haplotype with I133V, K419E, K421E, and F489L mutations in CYP51A, F129L-mutated Cyt b, and C-S135R mutation in the SDH-C subunit simultaneously (Table S2). *Ptt* with relevant mutations in all three fungicide target proteins simultaneously would be a high risk for efficient disease control when adapting and spreading in the population. Most of these isolates had a reduced sensitivity phenotype to mefentrifluconazole (median EC_50_ = 1.319 mg/L), fluxapyroxad (median EC_50_ = 0.171 mg/L), and azoxystrobin (median EC_50_ = 0.044 mg/L). However, sensitivity to the other tested fungicides remained high, with the median EC_50_ being 0.07 mg/L for bixafen, 0.021 mg/L for prothioconazole-desthio, and 0.004 mg/L for pyraclostrobin. Among these high-risk isolates, eight isolates in 2021 and four isolates in 2022 were collected from nearby fields and could have the same origin.

### 3.5. Pyrenophora teres *f.* teres Mating Types

The mating type analysis showed that both MAT-1 and MAT-2 are common in the Estonian *Ptt* population. In 2021, 80% of the isolates were MAT-2, and the population significantly differentiated (*p* < 0.001) from equal distribution of MAT-1 and MAT-2. However, in 2022, 52% of the isolates were MAT-2 and 48% were MAT-1; the ratio was nearly 1:1 in the population. Only MAT-1 was identified in 8% and 32% of the fields, and MAT-2 prevailed in 33% and 36% of the fields in years 2021 and 2022, respectively. Sexual reproduction can occur when opposite mating types meet on the same field site. Both *Ptt* mating types occurred together in 59% and 32% of the spring barley fields in 2021 and 2022, respectively.

## 4. Discussion

This is the first research study characterizing several molecular changes in fungicide target proteins (CYP51A, Cyt b, and SDH subunits C and D) and the *Cyp51A* gene promoter, which have a potential impact on the fungicide sensitivity of the *Ptt* field population in Northeastern Europe. Several *Ptt* isolates had reduced sensitivity to a new DMI active ingredient, mefentrifluconazole, influenced by target site mutations in the DMI target protein CYP51A and insert in the *Cyp51A* promoter region. The *Ptt* population was mostly sensitive to prothioconazole-desthio, bixafen, fluxapyroxad, pyraclostrobin, and azoxystrobin. In addition, the mating type analysis revealed that *Ptt* is expected to reproduce clonally in most of the fields, but possible sexual reproduction events may occur in the fields when both mating types meet.

The *Ptt* isolates, which had amino acid change phenylalanine to leucine in CYP51A in position 489, dominate in Estonia, 100% in 2021 and 79% in 2022. They are associated with reduced sensitivity to mefentrifluconazole, but not to prothioconazole-desthio. In a study from Australia, the same mutation was assumed to cause a considerable conformational rearrangement within the *Ptt* CYP51 protein haem cavity, resulting in a low inhibition effect of DMIs, such as tebuconazole, difenoconazole, and prochloraz [19]. In the Estonian *Ptt* population, two different codons gave rise to the phenylalanine to leucine amino acid change in position 489, from the wild-type TTC to either CTC or TTA (Table 2). In Australia, all the *Ptt* mutant isolates had an identical codon TTA for leucine [19], and the *Ptm* mutant isolates had three codons (TTA, TTG, and CTC) for leucine in amino acid position 489 in CYP51A [34]. The mutation F489L has been detected in Australia since 2013 in the *Ptt* population and since 2016 in the *Ptm* population [19,34], but in Europe, the *Cyp51A* mutation prevalence in *Ptt* populations is so far unknown.

The combination of mutations in *Cyp51A* with promoter insertions is contributing to the highest levels of DMI resistance in several cereal pathogen (e.g., *Z. tritici*, *Ptm*) populations [34,44]. Our finding of the 129 bp insertion element in the promoter of the *Cyp51A* of *Ptt* was noticed in isolates with reduced sensitivity to mefentrifluconazole, but their prothioconazole-desthio sensitivity remained high. Direct repeats of five base pairs (CATTT) on both ends of the 129 bp insertion are like 4–6 bp target site duplication sequences (TSDs) characterised in the case of LTR retrotransposons [45]. An identical insert with direct repeats was identified in moderately DMI-resistant *Ptm* isolates [34], but not in the *Ptt* isolates from Australia [19]. Moreover, highly DMI-resistant *Ptm* isolates had both an insertion element in the promoter and mutation F498L in CYP51A [34]. In the Estonian *Ptt* population, these molecular changes did not occur simultaneously in the same isolates.

However, sensitivity to prothioconazole-desthio was high in the Estonian *Ptt* population, and molecular changes in the target protein CYP51A and its promoter region did not reduce the sensitivity. A similar observation in the Australian *Ptt* population revealed high sensitivity to prothioconazole, but reduced sensitivity to other DMIs (tebuconazole, metconazole, and difenoconazole) in the same isolates [19]. Unfortunately, the data on DMI sensitivity of the *Ptt* population in Europe are not detailed and are limited to annual FRAC reports that show fluctuations in DMI sensitivity in France and Germany, but stable sensitivity in other European countries [46]. The marketed cereal plant protection products in Estonia most often contain prothioconazole as one active ingredient in combination with SDHI, amine, or other ingredients [17]. Although prothioconazole-containing products have been extensively applied to Estonian cereal fields for the last two decades, a significant sensitivity shift has not been noted in other cereal plant pathogen populations (*Z. tritici* and *R. collo-cygni*), regardless of the molecular changes in the CYP51 protein [47,48,49]. Furthermore, barley field trials in Northern Europe have shown satisfactory control of net blotch with 1 to 2 foliar applications of prothioconazole fungicide mixtures, even in high disease pressure (for example, in 2019) [6].

The Estonian *Ptt* population was mostly sensitive to the tested SDHIs fluxapyroxad and bixafen with a few exceptions. In the wild-type SDH enzyme, histidines at position 134 in subunits SDH-C and SDH-D coordinate the central iron atom of the haem b group. These two amino acid positions have been substituted to arginine in the case of SDHI insensitivity in *P. teres* [21]. Besides C-H134R and D-H134R, three further amino acid substitutions, C-N75S, C-G79R, and C-S135R, are clustered around haem b [21]. In the current study, out of these only mutations, C-S135R and D-H134R were identified in the *Ptt* population, and 30% of the *Ptt* isolates collected in 2021 and 40% in 2022 were mutated. Mutation B-H277Y, which has been found in other countries [21,36], was absent in the Estonian *Ptt* population. The fluxapyroxad and bixafen sensitivity were both affected in the SDH-mutated *Ptt* isolates in Estonia. In Rehfus et al.’s [21] study, the field dose of fluxapyroxad showed stable control for *P. teres* isolates with mutations leading to B-H277Y, C-N75S, C-S135R, D-D124N, and D-D145G. Higher resistance factors have been associated with mutations C-G79R, D-H134R, D-D124E, and C-H134R, with reduced fluxapyroxad efficacy [21,35]. In Argentina, the detection of *Ptt* isolates carrying double mutation C-N75S + D-D145G raises concerns in fluxapyroxad field efficacy [36]. As fluxapyroxad-containing products (e.g., Priaxor and Revytrex by BASF) are often used in cereal disease control and the spread of SDH-mutated *Ptt* isolates within the Estonian population is remarkable, the field efficacy should be monitored.

In general, the *Ptt* in Estonia was highly sensitive to QoI fungicides, and mutation F129L in Cyt b was identified in around 17% of the isolates and none had the G137R mutation. The target site mutation impacted azoxystrobin, but not pyraclostrobin sensitivity. The same effect was noted previously in another study, where pyraclostrobin was the most effective QoI fungicide, regardless of F129L mutation in the *Ptt* isolates [20]. Mutation F129L in *Ptt* Cyt b protein in Western Europe is frequent, and since the first notice of it in 2003, it rose to the frequency of 25% in 2014 [21]. *Ptt* population studies in Europe, Northern Africa, and Canada have shown variability but mostly high sensitivity to pyraclostrobin, azoxystrobin, and picoxystrobin [18,20,50]. Positively for disease control, it has been noted that mutation F129L does not reduce QoI sensitivity significantly, and these *Ptt* isolates can be controlled by recommended field rates of QoIs in vivo to almost the same extent as wild-type isolates [22,23]. In addition, *Ptt* cannot have mutation G143A in Cyt b, which has a high resistance factor and causes substantial disease control failure in several other cereal pathogen populations, for instance, *P. tritici-repentis* and *Z. tritici* [23,51,52].

Laboratory assays on new isolates with reduced sensitivity can be used as possible indicators of future changes in field performance [51]. The new less-sensitive isolates have a fitness advantage over wild-type strains under the selection pressure of fungicide [51]. In this study, 30 *Ptt* isolates had target site mutations in all three investigated fungicide target proteins, CYP51A, Cyt b, and SDH, and most of them had a reduced sensitivity phenotype to mefentrifluconazole, azoxystrobin, and fluxapyroxad. Although these isolates were mostly sensitive to other tested fungicides, it is critical to monitor and establish the spread of these mutated isolates prior to disease control failures.

In 2021, plant protection products Revystar XL, Revytrex (mefentrifluconazole, fluxapyroxad; BASF), and Balaya (mefentrifluconazole, pyraclostrobin; BASF) were marketed in Estonia for the first time, and farmers adopted these promptly into their cereal plant protection plans [17]. In Estonia, farmers are advised to apply these products once or twice in the active growing phases (BBCH 30-69) to protect spring barley against several plant diseases (net blotch, scald, ramularia leaf spot, powdery mildew, and leaf rust) and provide high quality and yield of the crop. Though mefentrifluconazole was not specifically developed to control *Ptt*, the active use of products containing mefentrifluconazole is a selection pressure for resistance development and needs further research if it selects for specific alterations in the CYP51 protein. As long as the Estonian *Ptt* population remains sensitive to SDHI agent fluxapyroxad and QoI agent pyraclostrobin, these products with a combination of two modes of action are efficient and provide sufficient control against net blotch, among other barley diseases.

*Ptt* is a seedborne pathogen, and gene flow or introduction of isolates with new traits (for example, target site mutations in fungicide target proteins) is possible through seed trade, then evolving and adapting to local environments, and through selection pressure of host cultivars and fungicide applications [12]. Farmers in Estonia import new batches of barley seed of various cultivars from Finnish, Danish, German, and other breeding companies, in addition to propagating seeds themselves. For instance, in Denmark and Germany, *Ptt* Cyt b protein F129L mutation levels are medium or medium to high [24]. SDH mutations C-G79R, C-H134R, C-N75S, and C-S135R occur most often in European populations [30]. Mutations in the SDH protein are absent in Finland but occur at low frequency in Denmark and high levels in Western Europe, including Germany [30].

*Ptt* has a mixed reproduction system, which contributes to the evolution of the pathogen [12]. Equal distribution of the two mating types is assumed in the absence of segregation bias and selection of one mating type [9]. In the present study, within the Estonian *Ptt* population in 2022, the MAT-1/MAT-2 ratio was 1:1, as expected in sexually reproducing population. Although several fields had only one mating type and sexual reproduction within these fields would be limited. In 2022, the sampling was more widespread compared to 2021 when MAT-1/MAT-2 ratio was 1:4 and one mating type dominated. *Ptt* population studies from Finland, Hungary, Italy, Australia, and Canada reported a 1:1 mating type ratio and possible sexual reproduction contributing to genetic diversity [9,53,54,55,56]. However, evidence from Dahanayaka et al. [54] showed deviation from the expected 1:1 ratio in population clusters from South Africa and Australia.

## 5. Conclusions

As this is the first detailed study of the current fungicide sensitivity of a widely distributed barley pathogen *Ptt* population in Estonia, it will serve as a baseline for future assessments. We identified multiple relevant molecular changes—F489L mutation in CYP51A, 129 bp insert in *Cyp51A* promoter, mutations C-S135R and D-H134R in SDH protein, and F129L mutation in Cyt b protein—affecting the sensitivity of the *Ptt* field population to fungicides of different modes of action. To prolong the effective lifetime of fungicides, they should be applied in alternation, as well as in mixtures where available, preferably including other modes of action. However, the selection of fungicide products is limited, and cereals as the main crops in Estonian commercial farms are generally protected with the same products. Thus, the selection pressure for fungicide resistance development in pathogen populations is strong. Barley seed trade may introduce new fungicide resistance-related mutations, and probable sexual reproduction in the *Ptt* population in Estonia could increase the genetic diversity and fitness of the pathogen. Therefore, it is crucial to continue with monitoring of the pathogen and its adaptation to fungicides to prevent disease management failures.

## Figures and Tables

**Table 1:** Oligonucleotides used for amplification and sequencing to confirm isolate species and detect point mutations in fungicide target genes, CYP51A promoter inserts, and mating type.

Target	Primer Name	Sequence (5’–3’)	Annealing Temp. (°C)	Amplicon Size (kb)	Reference
Fungal *ITS1-5.8S-ITS2* region	ITS1	TCCGTAGGTGAACCTGCGG	55	0.6	[38]
	ITS4	TCCTCCGCTTATTGATATGC			
*Ptt* form-specific	Ptt_R1ID1_F3	GACGCGGCTAATGTCGTAA	61	0.21	[35]
	Ptt_R1ID1_B3_R	GGCGGTAACAGCACCAAG			
MAT-1	Ptt-MAT1F	ATGAGACGCTAGTTCAGAGTCT	60	1.14	[39]
	Ptt-MAT1R	GATGCCCAGCCAAGGACAA			
MAT-2	Ptt-MAT2F	TACGTTGATGCAGCTTTCTCAAT	60	1.42	[39]
	Ptt-MAT2R	AACACCGTCCAAAGCACCT			
*sdhB*	KES 1825	CATAACCGAGGAAGCTTGAGTG	63	1.2	[21]
	KES 1837	CAAACACAACTCGCAATTAACGC			
*sdhC*	KES 1827	ATCACCCAACACCACCATCG	60	0.85	[21]
	KES 1828	ATGTTGCAAACTTCAATCGTACCC			
*sdhD*	KES 1833	CGATCCTTCAACCCACCTCCGA	60	0.75	[21]
	KES 1834	ACCCGCTTATGCATGCCACAG			
*Cyp51A*	PttCyp51A_1F	ATGCTCTCCCTCCTCTTCCTC	62	1.5	[19]
	PttCyp51A_3F ^a^	GCATTCCAACGTCGTCAAAG			
	PttCyp51A_3R	TTACCGCCTCTCCCAGC			
*cyt b* (F129L)	1PtcytB-F	TGGTGGGTGGCTGAATATGCTACT	58	0.35	[22]
	1PTcytB-R	CAGACATTCCAAGACTATTTGAGGAAC			
*cyt b* (G137R)	PttCytB-F2	GTGGTATAACCCGACAGG	58	0.35	current study
	PttCytB-R2	GCTATGAAATCAGCTTGCGCC			
*Cyp51A* promoter	PttCyp51A_Pro_F	GGCTCATAAATGGCGGAAC	58	0.57	[19]
	PttCyp51A_Pro_R	AGGAAGAGGAGGGAGAGCAT			
*Cyp51A* upstream region	PttCyp51A_Pro_F	GGCTCATAAATGGCGGAAC	55	1.3	[19]
	PttCyp51A_1R	GAGATCGTGGTACAGGCTTG			

^a^ Primer only for sequencing.

**Table 2:** The frequency of CYP51A haplotypes with specific amino acid substitutions identified in *Pyrenophora teres* f. *teres* population in 2021 and 2022.

Haplotype	Insert ^a^	CYP51A Amino Acid Polymorphism							Frequency (%)	
		I133V	G168D	L204V	M237I	K419E	K421E	F489L	2021	2022
Wild type ^b^	−	I	G	L	M	K	K	F	-	-
PttEE-A1	−	V	G	L	M	E	E	F	-	5.7
PttEE-A2	+	V	G	L	M	E	E	F	-	10
PttEE-A3	−	V	G	L	M	E	E	L ^c^	94.3	67.2
PttEE-A4	−	V	G	L	M	E	E	L ^d^	-	11.5
PttEE-14	−	V	G	L	I	E	E	F	-	2.1
PttEE-23	+	V	G	L	M	E	K	F	-	2.1
PttEE-4507	−	V	D	L	M	E	E	F	-	0.7
PttEE-4506	−	I	D	L	M	E	E	F	-	0.7
21PttEE-15	−	V	G	V	M	E	E	L	5.7	-

^a^ 129 bp insert present (+) in *Cyp51A* promoter. ^b^ *Ptt* CYP51A according to Mair et al. [19]. ^c^ Codon CTC. ^d^ Codon TTA.

**Table 3:** The frequency (%) of amino acid substitutions in SDHI and QoI fungicide target molecules and the frequency (%) of sensitive (S), reduced sensitivity (RS), and insensitive isolates (R) to SDHIs (fluxapyroxad and bixafen) and QoIs (azoxystrobin and pyraclostrobin) in *Pyrenophora teres* f. *teres* population in 2021 and 2022.

Year	Frequency of Amino Acid Substitutions			Fluxapyroxad ^a^			Bixafen ^a^		
	SDHB-H277Y	SDHC-S135R	SDHD-H134R	S	RS	R	S	RS	R
2021	0	30	0	62	36	2	94	6	0
2022	0	14	31	76	22	2	90	7	3
				**Azoxystrobin ^b^**			**Pyraclostrobin ^b^**		
	**CytB-F129L**	**CytB-G137R**		**S**	**RS**	**R**	**S**	**RS**	**R**
2021	30	0		63	37	0	78	18	4
2022	12	0		85	14	1	92	7	1

^a^ *Ptt* isolates were grouped according to SDHI sensitivity into sensitive (S, EC_50_ < 0.1 mg/L), reduced sensitivity (RS, EC_50_ = 0.1–1 mg/L), and insensitive isolates (R, EC_50_ > 1 mg/L). ^b^ *Ptt* isolates were grouped according to QoI sensitivity into sensitive (S, EC_50_ < 0.01 mg/L), reduced sensitivity (RS, EC_50_ = 0.01–1 mg/L), and insensitive isolates (R, EC_50_ > 1 mg/L).

## Data Availability

The DNA sequences of the *Ptt* fungicide target molecule genes with relevant single nucleotide mutations are made publicly available in the NCBI GenBank database with accession numbers OR530176, OR761967 to OR761973, and OR777246 to OR777248. Other raw data that support the findings of this study are available from the corresponding author upon reasonable request.

## Supplementary Materials

The following supporting information can be downloaded at: https://www.mdpi.com/article/10.3390/jof10040260/s1, Figure S1: Alignment of *Cyp51A* gene-promoter sequences. Insert-containing CYP51A promoter sequence from representative Estonian *Pyrenophora teres* f. *teres* isolate 22PTEE-0701 (*Ptt_07-01:* GenBank accession OR530176) compared to *Ptt* wild-type (*Ptt_W1:* GenBank accession HG992977) and *P. teres* f. *maculata* respective sequences (*Ptm_SG1…PTM_P7*: GenBank accessions OCTF02000001 and MT499783–MT499787) previously described in Mair et al. [34]. Sequence alignment was visualized using Jalview [43]; Table S1: Summary of Estonian *Pyrenophora teres* f. *teres* representative isolates and their GenBank accession numbers; Table S2: Collection of Estonian *Pyrenopora teres* f. *teres* isolates with relevant data of fungicide target gene sequences and GenBank accession number of representative isolates.
