# Supplementary material for: Fungicide Sensitivity Profile of Pyrenophora teres f. teres in Field Population
Source: J Fungi (Basel). 2024 Mar 29;10(4):260. doi: 10.3390/jof10040260 (PMC11051325; doi:10.3390/jof10040260)
Supplement: Supplementary file 1 [file jof-10-00260-s001.zip › Table S1.pdf]

Table S1. Summary of Estonian *Pyrenophora teres f. teres* representative isolates and their GenBank accession numbers.

Accession no	Representative isolate	Fungicide target protein gene	Insert in promoter	Position of relevant aa alteration
OR530176	22-PT-EE-36-02, 22-PT-EE-07-01	*Cyp51A*	+	F489 (TTC)
OR761967	22-PT-EE-35-05	*Cyp51A*	-	F489 (TTC)
OR761968	22-PT-EE-38-09	*Cyp51A*	-	489L (CTC)
OR761969	22-PT-EE-49-05	*Cyp51A*	-	489L (TTA)
OR761970	22-PT-EE-05-05	*sdhD*	NA	134R
OR761971	22-PT-EE-31-02	*sdhD*	NA	H134
OR761972	22-PT-EE-29-11	*sdhC*	NA	S135
OR761973	22-PT-EE-43-01	*sdhC*	NA	135R
OR777246	22-PT-EE-41-10	*cyt B*	NA	F129
OR777247	22-PT-EE-49-08	*cyt B*	NA	129L
OR777248	22-PT-EE-29-11	*sdhB*	NA	H277
