# Supplementary material for: Fungicide Sensitivity Profile of Pyrenophora teres f. teres in Field Population
Source: J Fungi (Basel). 2024 Mar 29;10(4):260. doi: 10.3390/jof10040260 (PMC11051325; doi:10.3390/jof10040260)
Supplement: Supplementary file 1 [file jof-10-00260-s001.zip › Table S2.pdf]

Table S2. Collection of Estonian *Pyrenopora teres f. teres* isolates with relevant data of fungicide target gene sequences and Genbank accession number of representative isolates.

Isolate[a]	County	Insert in CYP51A promoter	CYP51A[b]	Codon sequence in position 489 in CYP51A	SdhC	SdhD	Cyt b	GenBank Accession Number
22-PT-EE-05-01	Ida-Viru	-	PttEE-A3	CTC	-	D-H134R	-	-
22-PT-EE-05-02	Ida-Viru	-	PttEE-A3	CTC	-	D-H134R	-	-
22-PT-EE-05-03	Ida-Viru	-	PttEE-A3	CTC	-	D-H134R	-	-
22-PT-EE-05-04	Ida-Viru	-	PttEE-A3	CTC	-	D-H134R	-	-
22-PT-EE-05-05	Ida-Viru	-	PttEE-A3	CTC	-	D-H134R	-	OR761970 (*sdhD*)
22-PT-EE-05-06	Ida-Viru	-	PttEE-A3	CTC	-	D-H134R	-	-
22-PT-EE-05-07	Ida-Viru	-	PttEE-A3	CTC	-	D-H134R	-	-
22-PT-EE-05-08	Ida-Viru	-	PttEE-A3	CTC	-	D-H134R	-	-
22-PT-EE-05-09	Ida-Viru	-	PttEE-A3	CTC	-	D-H134R	-	-
22-PT-EE-05-10	Ida-Viru	-	PttEE-A3	CTC	-	D-H134R	-	-
22-PT-EE-07-01	Ida-Viru	+	PttEE-A2	TTC	-	D-H134R	-	OR530176 (*Cyp51A*)
22-PT-EE-07-02	Ida-Viru	+	PttEE-A2	TTC	-	D-H134R	-	-
22-PT-EE-07-03	Ida-Viru	-	PttEE-A3	CTC	-	-	-	-
22-PT-EE-07-04	Ida-Viru	-	PttEE-A3	CTC	-	-	-	-
22-PT-EE-09-01	Ida-Viru	-	PttEE-A3	CTC	-	-	-	-
22-PT-EE-09-02	Ida-Viru	-	PttEE-A3	CTC	-	-	-	-
22-PT-EE-09-03	Ida-Viru	-	PttEE-A3	CTC	-	-	-	-
22-PT-EE-09-04	Ida-Viru	-	PttEE-A3	CTC	-	-	-	-
22-PT-EE-09-05	Ida-Viru	-	PttEE-A3	CTC	-	-	-	-
22-PT-EE-11-01	Ida-Viru	-	PttEE-A3	CTC	-	-	-	-
22-PT-EE-11-02	Ida-Viru	-	PttEE-A3	CTC	-	-	-	-
22-PT-EE-11-03	Ida-Viru	-	PttEE-A3	CTC	-	-	-	-
22-PT-EE-16-03	Jõgeva	-	PttEE-A3	CTC	-	D-H134R	-	-
22-PT-EE-16-04	Jõgeva	-	PttEE-A3	CTC	-	D-H134R	-	-
22-PT-EE-16-09	Jõgeva	-	PttEE-A3	CTC	-	D-H134R	-	-

22-PT-EE-16-10	Jõgeva	-	PttEE-A3		CTC	-	D-H134R	-	-
22-PT-EE-23-01	Jõgeva	+	PttEE-A2	TTC		-	D-H134R	-	-
22-PT-EE-23-02	Jõgeva	+	PttEE-A2	TTC		-	D-H134R	-	-
22-PT-EE-23-03	Jõgeva	+	PttEE-A2	TTC		-	D-H134R	-	-
22-PT-EE-23-04	Jõgeva	+	PttEE-A2	TTC		-	D-H134R	-	-
22-PT-EE-23-05	Jõgeva	+	PttEE-23	TTC		-	D-H134R	-	-
22-PT-EE-23-06	Jõgeva	+	PttEE-23	TTC		-	D-H134R	-	-
22-PT-EE-02-01	Järva	-	PttEE-A3		CTC	-	-	-	-
22-PT-EE-13-01	Järva	-	PttEE-A3		CTC	-	-	-	-
22-PT-EE-13-02	Järva	-	PttEE-A3		CTC	-	-	-	-
22-PT-EE-13-03	Järva	-	PttEE-A3		CTC	-	-	-	-
22-PT-EE-13-04	Järva	-	PttEE-A3		CTC	-	-	-	-
22-PT-EE-13-05	Järva	-	PttEE-A3		CTC	-	-	-	-
22-PT-EE-13-06	Järva	-	PttEE-A3		CTC	-	-	-	-
22-PT-EE-13-07	Järva	-	PttEE-A3		CTC	-	-	-	-
22-PT-EE-14-01	Järva	-	PttEE-14	TTC		-	-	F129L	-
22-PT-EE-14-02	Järva	-	PttEE-14	TTC		-	-	F129L	-
22-PT-EE-14-03	Järva	-	PttEE-14	TTC		-	-	F129L	-
22-PT-EE-28-01	Lääne-Viru	-	PttEE-A3		CTC	-	-	-	-
22-PT-EE-28-02	Lääne-Viru	-	PttEE-A3		CTC	-	-	-	-
22-PT-EE-28-03	Lääne-Viru	-	PttEE-A3		CTC	-	-	-	-
22-PT-EE-28-04	Lääne-Viru	-	PttEE-A3		CTC	-	-	-	-
22-PT-EE-28-05	Lääne-Viru	-	PttEE-A3		CTC	-	-	-	-
22-PT-EE-29-01	Pärnu	-	PttEE-A3		CTC	-	-	-	-
22-PT-EE-29-02	Pärnu	-	PttEE-A3		CTC	-	-	-	-
22-PT-EE-29-03	Pärnu	-	PttEE-A3		CTC	-	-	-	-
22-PT-EE-29-04	Pärnu	-	PttEE-A3		CTC	-	-	-	-
22-PT-EE-29-05	Pärnu	-	PttEE-A3		CTC	-	-	-	-
22-PT-EE-29-06	Pärnu	-	PttEE-A3		CTC	-	-	-	-
22-PT-EE-29-07	Pärnu	-	PttEE-A3		CTC	-	-	-	-
22-PT-EE-29-08	Pärnu	-	PttEE-A3		CTC	-	-	-	-
22-PT-EE-29-09	Pärnu	-	PttEE-A3		CTC	-	-	-	-
22-PT-EE-29-10	Pärnu	-	PttEE-A3		CTC	-	-	-	-

22-PT-EE-29-11	Pärnu	-	PttEE-A3		CTC	-	-	-	OR761972 (*sdhC*), OR777248 (*sdhB*)
22-PT-EE-29-12	Pärnu	-	PttEE-A3		CTC	-	-	-	-
22-PT-EE-29-14	Pärnu	-	PttEE-A3		CTC	-	-	-	-
22-PT-EE-31-01	Pärnu	-	PttEE-A3		CTC	C-S135R	-	F129L	-
22-PT-EE-31-02	Pärnu	-	PttEE-A3		CTC	C-S135R	-	F129L	OR761971 (*sdhD*)
22-PT-EE-35-01	Tartu	-	PttEE-A1	TTC		-	D-H134R	-	-
22-PT-EE-35-02	Tartu	-	PttEE-A1	TTC		-	D-H134R	-	-
22-PT-EE-35-03	Tartu	-	PttEE-A1	TTC		-	D-H134R	-	-
22-PT-EE-35-04	Tartu	-	PttEE-A1	TTC		-	D-H134R	-	-
22-PT-EE-35-05	Tartu	-	PttEE-A1	TTC		-	D-H134R	-	OR761967 (*Cyp51A*)
22-PT-EE-35-06	Tartu	-	PttEE-A3		CTC	-	-	-	-
22-PT-EE-35-07	Tartu	-	PttEE-A3		CTC	-	-	-	-
22-PT-EE-35-08	Tartu	-	PttEE-A1	TTC		-	D-H134R	-	-
22-PT-EE-35-09	Tartu	-	PttEE-A1	TTC		-	D-H134R	-	-
22-PT-EE-35-10	Tartu	-	PttEE-A1	TTC		-	D-H134R	-	-
22-PT-EE-36-01	Tartu	+	PttEE-A2	TTC		-	D-H134R	-	-
22-PT-EE-36-02	Tartu	+	PttEE-A2	TTC		-	D-H134R	-	OR530176 (*Cyp51A*)
22-PT-EE-36-03	Tartu	+	PttEE-A2	TTC		-	D-H134R	-	-
22-PT-EE-36-04	Tartu	+	PttEE-A2	TTC		-	D-H134R	-	-
22-PT-EE-36-05	Tartu	+	PttEE-A2	TTC		-	D-H134R	-	-
22-PT-EE-36-06	Tartu	+	PttEE-23	TTC		-	D-H134R	-	-
22-PT-EE-36-07	Tartu	+	PttEE-A2	TTC		-	D-H134R	-	-
22-PT-EE-36-08	Tartu	+	PttEE-A2	TTC		-	D-H134R	-	-
22-PT-EE-36-09	Tartu	+	PttEE-A2	TTC		-	D-H134R	-	-
22-PT-EE-38-02	Tartu	-	PttEE-A3		CTC	C-S135R	-	-	-
22-PT-EE-38-05	Tartu	-	PttEE-A3		CTC	C-S135R	-	-	-
22-PT-EE-38-06	Tartu	-	PttEE-A3		CTC	C-S135R	-	F129L	-

22-PT-EE-38-07	Tartu	-	PttEE-A3	CTC		C-S135R	-	F129L	-
22-PT-EE-38-08	Tartu	-	PttEE-A4		TTA	C-S135R	-	F129L	-
22-PT-EE-38-09	Tartu	-	PttEE-A3	CTC		C-S135R	-	-	OR761968 (*Cyp51A*)
22-PT-EE-38-10	Tartu	-	PttEE-A3	CTC		C-S135R	-	-	-
22-PT-EE-04-01	Valga	-	PttEE-A3	CTC		-	-	-	-
22-PT-EE-41-01	Valga	-	PttEE-A3	CTC		-	-	-	-
22-PT-EE-41-02	Valga	-	PttEE-A3	CTC		-	-	-	-
22-PT-EE-41-03	Valga	-	PttEE-A3	CTC		-	-	-	-
22-PT-EE-41-04	Valga	-	PttEE-A3	CTC		-	-	-	-
22-PT-EE-41-05	Valga	-	PttEE-A3	CTC		-	-	-	-
22-PT-EE-41-06	Valga	-	PttEE-A3	CTC		-	-	-	-
22-PT-EE-41-07	Valga	-	PttEE-A3	CTC		-	-	-	-
22-PT-EE-41-08	Valga	-	PttEE-A3	CTC		-	-	-	-
22-PT-EE-41-09	Valga	-	PttEE-A3	CTC		-	-	-	-
22-PT-EE-41-10	Valga	-	PttEE-A3	CTC		-	-	-	OR777246 (*cytb*)
22-PT-EE-43-01	Valga	-	PttEE-A3	CTC		C-S135R	-	-	OR761973 (*SdhC*)
22-PT-EE-43-02	Valga	-	PttEE-A3	CTC		-	D-H134R	-	-
22-PT-EE-43-03	Valga	-	PttEE-A3	CTC		-	D-H134R	-	-
22-PT-EE-43-04	Valga	-	PttEE-A3	CTC		-	D-H134R	-	-
22-PT-EE-43-05	Valga	-	PttEE-A3	CTC		-	D-H134R	-	-
22-PT-EE-03-02	Viljandi	-	PttEE-A3	CTC		-	-	-	-
22-PT-EE-03-03	Viljandi	-	PttEE-A3	CTC		-	-	-	-
22-PT-EE-03-04	Viljandi	-	PttEE-A3	CTC		-	-	-	-
22-PT-EE-03-05	Viljandi	-	PttEE-A3	CTC		-	-	-	-
22-PT-EE-03-06	Viljandi	-	PttEE-A3	CTC		-	-	-	-
22-PT-EE-03-07	Viljandi	-	PttEE-A3	CTC		-	-	-	-
22-PT-EE-01-01	Võru	-	PttEE-A3	CTC		-	-	-	-
22-PT-EE-01-02	Võru	-	PttEE-A3	CTC		-	-	-	-
22-PT-EE-01-03	Võru	-	PttEE-A3	CTC		-	-	-	-
22-PT-EE-01-04	Võru	-	PttEE-A3	CTC		-	-	-	-
22-PT-EE-01-05	Võru	-	PttEE-A3	CTC		-	-	-	-
22-PT-EE-01-06	Võru	-	PttEE-A3	CTC		-	-	-	-
22-PT-EE-01-07	Võru	-	PttEE-A3	CTC		-	-	-	-
22-PT-EE-01-08	Võru	-	PttEE-A3	CTC		-	-	-	-
22-PT-EE-45-01	Võru	-	PttEE-A3	CTC		-	-	-	-
22-PT-EE-45-05	Võru	-	PttEE-A3	CTC		-	-	-	-

22-PT-EE-45-06	Võru	-	PttEE-4506	TTC			-	-	-	-
22-PT-EE-45-07	Võru	-	PttEE-4507	TTC			-	-	-	-
22-PT-EE-45-08	Võru	-	PttEE-A3		CTC		-	-	-	-
22-PT-EE-49-01	Võru	-	PttEE-A3		CTC		C-S135R	-	F129L	-
22-PT-EE-49-04	Võru	-	PttEE-A4			TTA	C-S135R	-	F129L	-
22-PT-EE-49-05	Võru	-	PttEE-A4			TTA	C-S135R	-	F129L	OR761969 (*Cyp51A*)
22-PT-EE-49-06	Võru	-	PttEE-A4			TTA	C-S135R	-	F129L	-
22-PT-EE-49-07	Võru	-	PttEE-A4			TTA	C-S135R	-	F129L	-
22-PT-EE-49-08	Võru	-	PttEE-A4			TTA	C-S135R	-	F129L	OR777247 (*cytb*)
22-PT-EE-49-09	Võru	-	PttEE-A4			TTA	C-S135R	-	F129L	-
22-PT-EE-49-10	Võru	-	PttEE-A4			TTA	C-S135R	-	F129L	-
22-PT-EE-49-11	Võru	-	PttEE-A4			TTA	C-S135R	-	F129L	-
22-PT-EE-50-01	Võru	-	PttEE-A4			TTA	-	-	-	-
22-PT-EE-50-02	Võru	-	PttEE-A4			TTA	-	-	-	-
22-PT-EE-50-03	Võru	-	PttEE-A4			TTA	-	-	-	-
22-PT-EE-50-04	Võru	-	PttEE-A4			TTA	-	-	-	-
22-PT-EE-50-05	Võru	-	PttEE-A4			TTA	-	-	-	-
22-PT-EE-50-06	Võru	-	PttEE-A4			TTA	-	-	-	-
22-PT-EE-50-07	Võru	-	PttEE-A4			TTA	-	-	-	-
21-PT-EE-04-02	Ida-Viru	-	21PttEE-15		CTC		-	-	-	-
21-PT-EE-04-05	Ida-Viru	-	PttEE-A3		CTC		-	-	-	-
21-PT-EE-04-06	Ida-Viru	-	PttEE-A3		CTC		-	-	-	-
21-PT-EE-04-07	Ida-Viru	-	PttEE-A3		CTC		-	-	-	-
21-PT-EE-05-03	Ida-Viru	-	PttEE-A3		CTC		-	-	-	-
21-PT-EE-05-04	Ida-Viru	-	PttEE-A3		CTC		-	-	-	-
21-PT-EE-05-05	Ida-Viru	-	PttEE-A3		CTC		-	-	-	-
21-PT-EE-05-06	Ida-Viru	-	PttEE-A3		CTC		-	-	-	-
21-PT-EE-05-07	Ida-Viru	-	PttEE-A3		CTC		-	-	-	-
21-PT-EE-18-01	Ida-Viru	-	PttEE-A3		CTC		-	-	-	-
21-PT-EE-18-03	Ida-Viru	-	PttEE-A3		CTC		-	-	-	-
21-PT-EE-18-04	Ida-Viru	-	21PttEE-15		CTC		-	-	-	-
21-PT-EE-18-05	Ida-Viru	-	PttEE-A3		CTC		-	-	-	-
21-PT-EE-03-01	Jõgeva	-	PttEE-A3		CTC		-	-	-	-
21-PT-EE-03-02	Jõgeva	-	PttEE-A3		CTC		-	-	-	-
21-PT-EE-03-03	Jõgeva	-	PttEE-A3		CTC		-	-	-	-
21-PT-EE-03-06	Jõgeva	-	PttEE-A3		CTC		-	-	-	-

21-PT-EE-06-01	Lääne-Viru	-	PttEE-A3	CTC	-	-	-	-
21-PT-EE-06-04	Lääne-Viru	-	PttEE-A3	CTC	-	-	-	-
21-PT-EE-06-06	Lääne-Viru	-	PttEE-A3	CTC	-	-	-	-
21-PT-EE-11-01	Saare	-	PttEE-A3	CTC	-	-	-	-
21-PT-EE-11-02	Saare	-	PttEE-A3	CTC	-	-	-	-
21-PT-EE-11-03	Saare	-	PttEE-A3	CTC	-	-	-	-
21-PT-EE-11-04	Saare	-	PttEE-A3	CTC	-	-	-	-
21-PT-EE-07-01	Tartu	-	PttEE-A3	CTC	C-S135R	-	F129L	-
21-PT-EE-07-02	Tartu	-	PttEE-A3	CTC	C-S135R	-	F129L	-
21-PT-EE-07-03	Tartu	-	PttEE-A3	CTC	C-S135R	-	F129L	-
21-PT-EE-07-04	Tartu	-	PttEE-A3	CTC	C-S135R	-	F129L	-
21-PT-EE-07-05	Tartu	-	PttEE-A3	CTC	C-S135R	-	F129L	-
21-PT-EE-07-06	Tartu	-	PttEE-A3	CTC	C-S135R	-	F129L	-
21-PT-EE-07-07	Tartu	-	PttEE-A3	CTC	C-S135R	-	F129L	-
21-PT-EE-07-08	Tartu	-	PttEE-A3	CTC	C-S135R	-	F129L	-
21-PT-EE-08-01	Tartu	-	PttEE-A3	CTC	-	-	-	-
21-PT-EE-08-02	Tartu	-	PttEE-A3	CTC	-	-	-	-
21-PT-EE-08-03	Tartu	-	PttEE-A3	CTC	-	-	-	-
21-PT-EE-08-04	Tartu	-	PttEE-A3	CTC	-	-	-	-
21-PT-EE-09-02	Tartu	-	PttEE-A3	CTC	-	-	-	-
21-PT-EE-09-03	Tartu	-	PttEE-A3	CTC	-	-	-	-
21-PT-EE-09-04	Tartu	-	PttEE-A3	CTC	-	-	-	-
21-PT-EE-15-01	Võru	-	PttEE-A3	CTC	C-S135R	-	F129L	-
21-PT-EE-15-02	Võru	-	PttEE-A3	CTC	C-S135R	-	F129L	-
21-PT-EE-15-03	Võru	-	21PttEE-15	CTC	C-S135R	-	F129L	-
21-PT-EE-15-04	Võru	-	PttEE-A3	CTC	C-S135R	-	F129L	-
21-PT-EE-15-05	Võru	-	PttEE-A3	CTC	C-S135R	-	F129L	-
21-PT-EE-15-06	Võru	-	PttEE-A3	CTC	C-S135R	-	F129L	-
21-PT-EE-15-07	Võru	-	PttEE-A3	CTC	C-S135R	-	F129L	-
21-PT-EE-15-08	Võru	-	PttEE-A3	CTC	C-S135R	-	F129L	-

21-PT-EE-16-01	Võru	-	PttEE-A3	CTC	-	-	-	-
21-PT-EE-16-02	Võru	-	PttEE-A3	CTC	-	-	-	-
21-PT-EE-16-05	Võru	-	PttEE-A3	CTC	-	-	-	-
21-PT-EE-16-06	Võru	-	PttEE-A3	CTC	-	-	-	-
21-PT-EE-16-08	Võru	-	PttEE-A3	CTC	-	-	-	-
21-PT-EE-16-10	Võru	-	PttEE-A3	CTC	-	-	-	-

[a] Isolate number starting with 21 or 22 indicates collection year 2011 or 2022, respectively.

[b] types of CYP51A haplotype according to this study, Table 2.
