# Supplementary figures and images for: Fungicide Sensitivity Profile of Pyrenophora teres f. teres in Field Population
Source: J Fungi (Basel). 2024 Mar 29;10(4):260. doi: 10.3390/jof10040260 (PMC11051325; doi:10.3390/jof10040260)

# insert (129 bp)

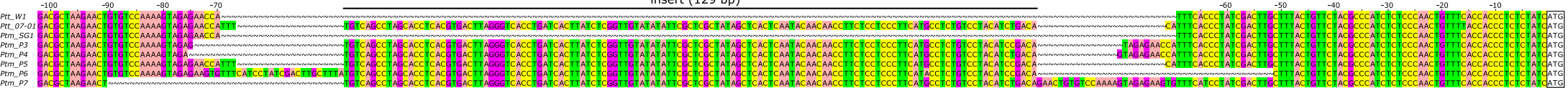

Supplement: Supplementary file 1 [file jof-10-00260-s001.zip › Figure S1.pdf]
